# Bilateral Deficit in Explosive Force Production Is Not Caused by Changes in Agonist Neural Drive

**DOI:** 10.1371/journal.pone.0057549

**Published:** 2013-03-05

**Authors:** Matthew W. Buckthorpe, Matthew T. G. Pain, Jonathan P. Folland

**Affiliations:** 1 School of Sport, Exercise and Health Sciences, Loughborough University, Loughborough, Leicestershire, United Kingdom; 2 Isokinetic Medical Group, London, United Kingdom; Universidad Europea de Madrid, Spain

## Abstract

Bilateral deficit (BLD) describes the phenomenon of a reduction in performance during synchronous bilateral (BL) movements when compared to the sum of identical unilateral (UL) movements. Despite a large body of research investigating BLD of maximal voluntary force (MVF) there exist a paucity of research examining the BLD for explosive strength. Therefore, this study investigated the BLD in voluntary and electrically-evoked explosive isometric contractions of the knee extensors and assessed agonist and antagonist neuromuscular activation and measurement artefacts as potential mechanisms. Thirteen healthy untrained males performed a series of maximum and explosive voluntary contractions bilaterally (BL) and unilaterally (UL). UL and BL evoked twitch and octet contractions were also elicited. Two separate load cells were used to measure MVF and explosive force at 50, 100 and 150 ms after force onset. Surface EMG amplitude was measured from three superficial agonists and an antagonist. Rate of force development (RFD) and EMG were reported over consecutive 50 ms periods (0–50, 50–100 and 100–150 ms). Performance during UL contractions was compared to combined BL performance to measure BLD. Single limb performance during the BL contractions was assessed and potential measurement artefacts, including synchronisation of force onset from the two limbs, controlled for. MVF showed no BLD (P = 0.551), but there was a BLD for explosive force at 100 ms (11.2%, P = 0.007). There was a BLD in RFD 50–100 ms (14.9%, P = 0.004), but not for the other periods. Interestingly, there was a BLD in evoked force measures (6.3–9.0%, P<0.001). There was no difference in agonist or antagonist EMG for any condition (P≥0.233). Measurement artefacts contributed minimally to the observed BLD. The BLD in volitional explosive force found here could not be explained by measurement issues, or agonist and antagonist neuromuscular activation. The BLD in voluntary and evoked explosive force might indicate insufficient stabiliser muscle activation during BL explosive contractions.

## Introduction

Bilateral deficit (BLD) has been used to describe the phenomenon of a reduction in performance during synchronous bilateral (BL) movements when compared to the sum of identical unilateral (UL) movements. A large body of research concerning BLD has been conducted using isometric and isokinetic tests of maximal voluntary force (MVF) production (for a review see [1]). BLD and has been reported with deficits of up to ∼25% [2]–[4], and therefore represents a potentially influential factor in the expression of BL muscle strength. However, explosive strength is often considered functionally more important than MVF during explosive movements, such as sprinting and jumping or restabilising the body following a loss of balance [5]–[8]. There is though, a paucity of research examining BLD in explosive strength with equivocal findings and limited mechanistic evidence. A BLD in peak rate of force development (RFD) has been reported to range between 0–24% [3], [9], [10], [11], with some studies indicating a greater BLD in RFD than MVF [9], [11], whereas others have not [3], [11].

Despite a large body of research examining BLD, the exact mechanisms explaining the phenomenon are unresolved. The primary explanation put forward for BLD during maximum isometric and isokinetic contractions is reduced neural drive to the agonist muscles. However, the evidence is equivocal, with several studies documenting parallel reductions in force and agonist activation during bilateral tasks [12], [13] whereas others have not [2], [11], [14], [15]. In the context of explosive strength, agonist activation has been found to be an important determinant of explosive force production [6], [7], [16]. Therefore, explosive force may be more susceptible to any reduction in agonist neural drive than MVF, and thus a more pronounced BLD for explosive than MVF could be expected. However, during the explosive phase of BL vs. UL contractions only one study has assessed agonist, and none have documented antagonist, neuromuscular activation. Van Dieen et al. [9] reported no change in agonist activation, despite a 13% decline in peak RFD.

The equivocal evidence for agonist activation contributing to a BLD in MVF might relate to the sensitivity of EMG measures, which have been questioned for their ability to detect small differences [10]. The absolute EMG amplitude is influenced by a multitude of intrinsic and extrinsic factors that are unrelated to the level of muscle activation [17]. Normalisation of the surface EMG amplitude to a maximal compound muscle action potential (*M*_max_) is considered a more sensitive measurement tool, but it has not previously been used to investigate the mechanistic basis of any BLD. The assessment of evoked explosive contractions can give insight into the capacity of the muscle-tendon unit (MTU) for explosive force production without the influence of voluntary commands. Identification of BLD in electrically evoked force would indicate BLD mechanism(s) exclusive of voluntary neural drive to the agonist muscles. However, the possibility of a BLD in evoked force production has not been investigated. Furthermore the comparison of volitional to evoked explosive force may also provide an alternative measure of the volitional neural efficacy.

Other potential mechanisms not previously considered that may contribute to a BLD in explosive strength include methodological artefacts associated with the measurement of BLD. For example, a BLD in explosive voluntary force could be due to a lack of synchronisation of agonist activation and force onset from the two limbs. Any offset or delay in the activation and force development from the second limb could compromise combined BL performance and contribute to BLD even if performance of each individual limb in this BL situation were equivalent to UL performance. An additional potential contributory factor arises from the fact that investigators typically utilise a small number of UL and BL contractions, and take the best UL and BL contractions for analysis and comparison (e.g. [10], [11]). However, this comparison may involve a statistical bias in favour of UL performance. BL performance relies on the simultaneous performance of two limbs, and statistically it is unlikely that both limbs will produce their highest UL performance during the same BL contraction. This simple measurement artefact could contribute to any apparent BLD irrespective of any physiological effects. Furthermore, as explosive force/RFD is less reliable than MVF [18], this measurement artefact might exert a greater bias on the BLD during explosive contractions. Essentially, whilst combined BL performance (i.e. the best effort of both legs when measured together) is clearly the actual and criterion measure of BL capability, due to possible measurement artefacts it may under represent the best effort of either leg in the BL situation. Comparison of UL performance to both combined BL performance, and performance of each limb during BL contractions, may highlight the influence measurement artefacts.

The aim of the study was to assess whether a BLD exists in voluntary and evoked explosive force production of the knee extensors, and document the contribution of agonist and antagonist neuromuscular activation, as well as measurement issues to any BLD in voluntary explosive force production. It was hypothesised that there would be a more substantial BLD for explosive force/RFD than MVF. This could be due to a more pronounced reduction in agonist neuromuscular activation and a stronger influence of methodological factors during explosive than maximum voluntary contractions.

## Methods

### Participants

Twelve healthy asymptomatic male participants completed the study (mean ± sd: age, 23.9±3.7 yr; height, 168.8±31.4 cm; body mass, 77.3±6.9 kg). Data from previously published research [9] was used to estimate the effect size for estimated BLD of explosive force/RFD. Cautiously, we aimed to detect a standardized effect size of 1.1. This standardized effect size, a statistical power of 80% (1– β = 0.80) and α = 0.05 were used to determine the necessary sample size of 11 participants. The participants were recreationally active (up to three activity sessions per week), but had not been involved in any systematic physical training during the preceding 12 months. All participants provided written informed consent prior to their involvement in the study, which complied with the Declaration of Helsinki and was approved by the Ethical Advisory Committee of Loughborough University.

### Overview

Participants attended the laboratory on two separate occasions, once for familiarisation and then for a main trial one week later. The two trials involved the same protocol and were completed at a consistent time of day. The main session involved the measurement of force and surface EMG during a series of voluntary (maximal and explosive) and electrically-evoked (twitch and octet) contractions of the knee extensors performed during either UL or BL contractions. In addition UL knee flexor maximum voluntary contractions (MVCs) with each leg were also performed for normalisation of antagonist EMG. To control for the influence of possible order effects, the order of voluntary contractions, involved first UL contraction(s) (either dominant or non-dominant leg, contraction order was randomly assigned), then BL contraction(s), and finally UL contraction(s) with the remaining limb (i.e. UL-BL-UL). Evoked measures began with the same limb that commenced the voluntary contractions followed by UL contractions of the remaining limb, and finally BL contractions (UL-UL-BL). Electrically evoked contractions can cause discomfort, and are not tolerated well by all participants. Therefore, it was decided to elicit single twitch and octet (8 pulses at 300 Hz) contractions unilaterally on both legs first before BL contractions to ensure as many participants completed the evoked measures as possible. In order to assess the BLD of voluntary and evoked contractions, performance during UL contractions were averaged and compared to the genuine BL performance, which involved the simultaneous averaged performance of both limbs obtained from a mutual onset during the same BL contractions (BL_BL_). Furthermore, the contribution of methodological artefacts (e.g. synchronisation of force onset) was also assessed. This involved comparing UL contractions with single limb performance measured during BL contractions. In practice this was facilitated by the discrete recording (i.e. two independent force transducers) and analysis (i.e. separate force onset) of each limb during BL efforts before averaging across both limbs (BL_UL_). Thus, allowing for assessment of UL vs. BL_UL_ without the potentially confounding influence of methodological artefacts.

### Force Measurement

Participants were firmly secured in a custom built strength testing chair with straps across the pelvis and shoulders to minimise extraneous movement. The hip and knee angles were fixed at 100 and 120° (full extension = 180°), respectively. An ankle strap was placed 2 cm proximal to the medial malleolus of each limb in series with two separate S-Beam tension/compression load cells (one for each limb, linear response up to 1500 N, Force Logic UK, Berkshire, UK) positioned perpendicular to tibial movement. The force signal was amplified (x500) and interfaced with an analogue to digital converter (CED micro 1401, CED, Cambridge, UK) and sampled at 2000 Hz with a PC utilising Spike 2 software (CED, Cambridge, UK). Real-time biofeedback of the force response was provided on a computer monitor. During off-line analysis the force signals were notch filtered at 50 Hz (to remove mains harmonics) and low pass filtered at 500 using a fourth order zero-lag Butterworth digital filter.

### Electrical Stimulation

The femoral nerve of each leg was electrically stimulated (via two constant current, variable voltage stimulators; DS7AH, Digitimer Ltd., UK) with square wave pulses (0.2 ms in duration) to elicit i) single twitch contractions and ii) octet contractions (8 pulses at 300 Hz) to determine the muscle’s maximal capacity for RFD. An anode (carbon rubber electrode, 7×10 cm; Electro-Medical Supplies, Greenham, UK) was taped to the skin over the greater trochanter of each limb. A cathode was taped to the skin over the femoral nerve in the femoral triangle of each leg. Both cathodes were identical custom-adapted stimulation probes 1 cm in diameter (Electro-Medical Supplies, Wantage, UK) which protruded 2 cm perpendicular from the centre of a plastic base (4×5 cm). The precise location of the each cathode was determined as the position which elicited the greatest twitch response for a particular submaximal current during UL contractions. During BL evoked contractions both stimulators were triggered simultaneously via the Spike 2 software.

### Surface Electromyography (EMG)

Surface EMG was recorded from the superficial quadriceps [rectus femoris (RF), vastus lateralis (VL) and vastus medialis (VM)] and a knee flexor [bicep femoris (BF)] of both legs using two Delsys Bagnoli-4 EMG systems (Delsys, Boston, USA). Following preparation of the skin (shaving, lightly abrading and cleansing with 70% ethanol), double differential electrodes (1 cm inter-electrode distance, DE-3.1, Delsys) were attached over each muscle using adhesive interfaces. To normalise the placement across individuals, the electrodes were positioned in the centre of the muscle belly parallel to the presumed orientation of the muscle fibers at specific lengths along the thigh (from the lateral epicondyle of the femur to the greater trochanter: VM, 25%; VL, 50%; RF, 60%; BF, 50%). The reference electrode was placed on the patella of the same limb. EMG signals were amplified (x1000; differential amplifier, 20–450 Hz) and synchronised with force data by recording at 2000 Hz with the same analogue to digital converter, PC and software (Spike 2) as the force signal. During off-line analysis the EMG signals were band-pass filtered between 6 and 500 Hz using a 4^th^ order zero-lag Butterworth digital filter.

### Protocol

#### Explosive voluntary contractions

**Figure 1:**
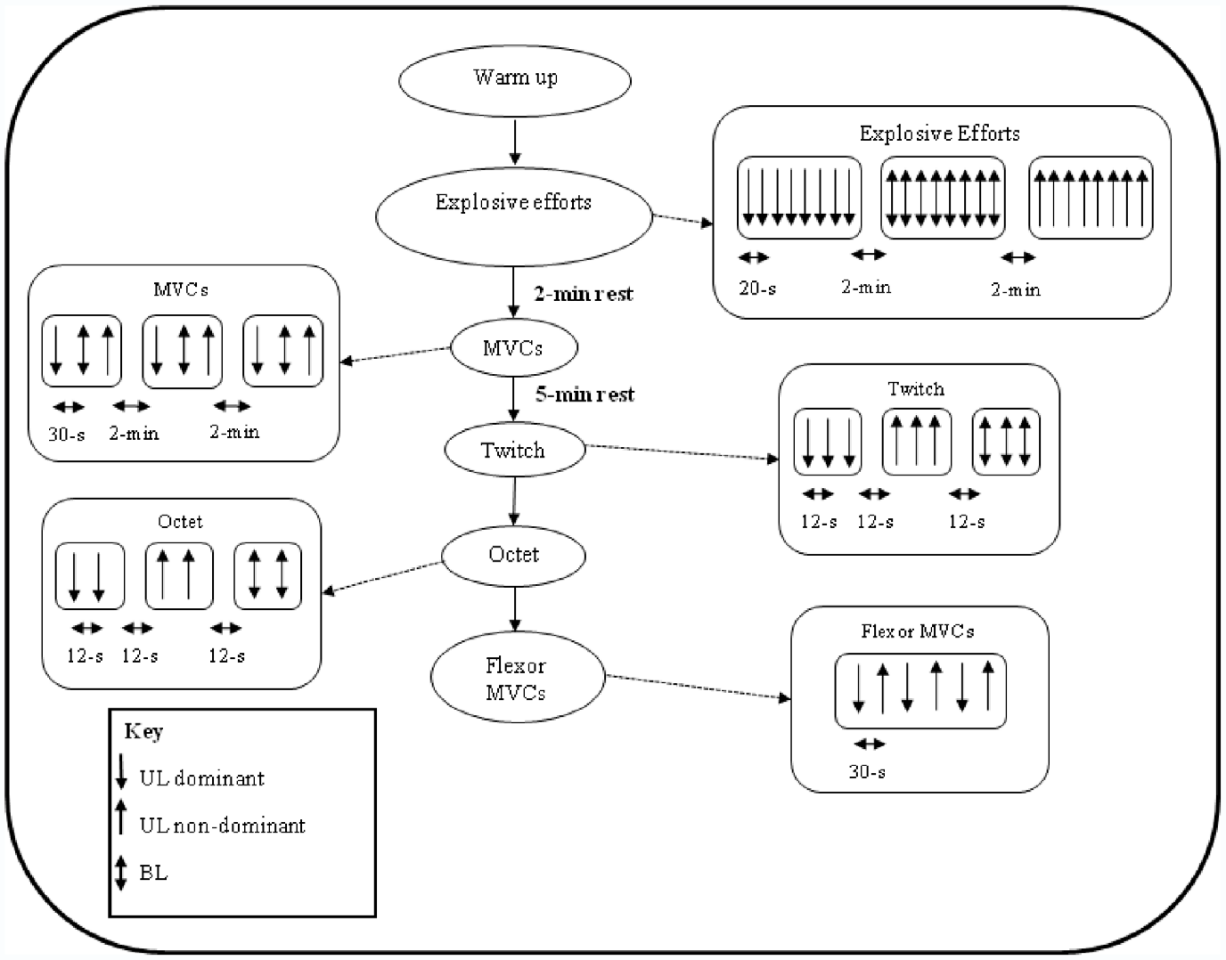
Schematic diagram of the protocol.

Once the participants were firmly secured in the testing chair they performed a warm-up, which consisted of two UL (with each limb) and BL contractions of the knee extensors at 50 and 75% presumed MVF. Participants then performed eight successful explosive voluntary contractions (separated by 20 s rest) of each contraction type (UL-BL-UL, with 2 min between each series, see figure 1). For each contraction participants were instructed to extend their knee(s) as ‘fast’ and as hard as possible for ∼1 s from a relaxed state [11]. Contractions that had any pre-tension or countermovement were discarded and another attempt was made. To determine if a countermovement or pre-tension had occurred, the resting force level was displayed on a sensitive scale. The slope of the force time curve (10 ms time constant) was displayed throughout testing and the peak slope was used to provide visual performance feedback to participants after each contraction. Furthermore, participants were required to exceed 80% MVF during these explosive contractions [7], [18] specific to that leg(s) which was depicted with a horizontal cursor on the screen. For the BL explosive contractions identical criteria and feedback were used based on the averaged force signal from both load cells.

The three contractions in each condition with the highest peak slope and no discernible countermovement or pre-tension (change in force of <0.5 N in the preceding 100 ms) were used for analysis. We have previously demonstrated that using the best three contractions from a series of explosive voluntary contractions following sufficient familiarisation provides reliable group explosive force and EMG measures (see [18]). Force and EMG measurements were taken at specific time points/periods and all measurements were averaged across these three contractions. Signal onsets of all voluntary and evoked contractions were visually identified [19]–[22] according to previous methods from our laboratory (see [7], [18]). Force was measured at 50, 100, and 150 ms (defined as F_50_, F_100_, F_150_), from the onset of contraction. RFD was measured over three consecutive 50 ms time periods from the onset of force (RFD_0­50_, RFD_50­100_, RFD_100­150_). For evaluating purely BL performance (i.e. the average combined ability of the two legs, BL_BL_) force onset was defined as the deflection of the averaged force signal from baseline. However for assessing UL performance during BL efforts (BL_UL_) force onsets were specific to that leg. EMG signal amplitude was quantified as the RMS measured in consecutive windows 0–50, 50–100, and 100–150 ms from the onset of EMG activity in the first agonist muscle to be activated within that limb(s). EMG from each agonist muscle was normalised to the peak-to-peak amplitude of a maximum compound action potential (*M*_max_) of that muscle during UL contractions (see below) and averaged across the three superficial quadriceps muscles to give a mean value for the quadriceps. EMG from the BF was normalised to EMG at knee flexor MVF (see below) of that muscle (Antagonist EMG). Although, the study was a within session design, the EMG was normalised to reduce the between subject variation [18] which would be expected to increase the effect size and power of statistical comparisons between the conditions. EMG onsets were identified from the first agonist muscle to be activated specific to each leg during UL and BL_UL_ conditions and the first muscle to be activated irrespective of the leg during the BL_BL_ condition. Additionally, the difference between the onsets of force of the two limbs in the BL contractions was identified. The time between the first agonist muscle to be activated and onset of force was determined as the maximum electromechanical delay (EMD*_max_*).

#### Maximum voluntary Contractions

Following two minutes rest, participants performed three sets of a single MVC of each type in the specified order (UL-BL-UL) with ≥30 s between MVCs and 2 min between sets. For each MVC they were instructed to push as hard as possible for 3 s with biofeedback and verbal encouragement provided during and between each maximal contraction. Knee extensor maximal voluntary force (MVF) was the greatest instantaneous force achieved by the participant in any of the MVCs specific to each condition. The root mean square (RMS) of the EMG signal for each muscle (RF, VM, VL and BF) was calculated over a 500 ms epoch surrounding MVF (250 ms either side). Each individual agonist muscle EMG was normalised to *M*_max_ (see below) before averaging across the three muscles to provide a mean value for the quadriceps (Agonist EMG). BF EMG was expressed as a percentage of BF EMG at knee flexor MVF (see below).

#### Electrically-evoked twitch and octet contractions

Five minutes separated the MVCs and evoked measurements. Evoked measures began with the same limb that commenced the voluntary contractions followed by UL contractions of the remaining limb, and finally BL contractions (UL-UL-BL). Twitch contractions were elicited at incremental current intensities until a simultaneous plateau in the force and *M*-wave response was observed. Thereafter, the current was increased by 20% and three supra-maximal twitches were elicited (separated by 12 s) for each limb during UL contractions. For BL contractions, the current was reduced, and incremental (25, 50, 75% of the supramaximal current used during UL contractions specific to that limb) evoked contractions were elicited, and three supramaximal BL twitch contractions were recorded. Two participants withdrew from the twitch measurements, therefore twitch responses are reported for N = 10. The mean *M*_max_ of these three supramaximal *M*-waves was determined for each muscle and used for normalisation purposes. The twitch force response was assessed at 50 ms after force onset (F_50_), peak force (PF), and pRFD (10 ms time constant) and averaged across the three contractions for each performance measure.

For the evoked octet contractions the current was once again reduced and step wise increments were delivered 15 s apart until the same supramaximal current intensity was achieved (typically 4–5 increments were performed). Two maximal evoked octet contractions were then elicited. The order of contractions was the same as during evoked twitch contractions (i.e. UL-UL-BL). Three participants withdrew from the octet measurements, therefore octet responses are reported for N = 9. During analysis, the average of the two octet contractions for each contraction type was taken. Analysis included measurement of force at 50 ms (F_50_), PF and pRFD. As an additional measure of overall neural efficacy, voluntary F_50_ for the three different measurements was reported as a percentage of the equivalent octet F_50_ to assess the participant’s voluntary activation capacity over the initial 50 ms of the contraction [8], [23].

#### Knee flexor MVCs

Following a series of submaximal knee flexor contractions (25, 50 and then 75% predicted MVF), participants performed three alternating UL MVCs of each leg. Each efforts was separated by ≥30 s in which participants were instructed to pull as hard as possible for 3 s with biofeedback and verbal encouragement during and between each maximal contraction. Knee flexor (BF) RMS EMG was assessed with a 500 ms RMS epoch around knee flexor MVF (250 ms either side, EMG_max_) and used for normalisation of antagonist EMG during maximal and explosive knee extensor contractions. Four participants had very low BF signal to noise ratios and therefore antagonist EMG data was reported for N = 8.

### Statistical Analysis

Data are reported as mean ± standard deviation (SD). One-way ANOVA was used to identify significant differences between voluntary performance measures across the three conditions (UL vs. BL_BL_ vs. BL_UL_). In the event of significant differences, paired t-tests were performed. For indices measured at two or more time points (EMG, force, RFD during explosive contractions) the effect of test condition (UL vs. BL_BL_ vs. BL_UL_) was analysed using a two-way repeated measures ANOVA (condition [3] × time [3]). Pairwise comparisons with Bonferroni correction were performed to locate the difference between test conditions at specific time points. BLD was defined as a difference between the BL_BL_ and UL conditions. Prior to performing the statistical analysis, confirmation of data normality was performed using Shapiro-Wilk test of normality. Statistical analysis was performed using SPSS version 19 and statistical significance was set at P<0.05.

## Results

### Voluntary Contractions

There was no difference in MVF (ANOVA, P = 0.551, Table 1) or agonist (ANOVA, P = 0.269, Table 1) or antagonist (ANOVA, P = 0.987, Table 1) EMG at MVF between the three measurement conditions.

**Table 1:** Force and EMG during maximum voluntary contractions performed unilaterally (UL) and bilaterally (BL_BL_, averaged simultaneous performance of both limbs; BL_UL_, single leg performance during BL contractions).

	UL	BL_BL_	BL_UL_
MVF (N)	736±83	739±92	744±89
Agonist EMG (%*M*_max_)	8.2±2.0	8.6±2.5	8.3±2.3
Antagonist EMG (%EMG_max_)	8.4±6.8	8.5±5.1	8.9±6.4

MVF, Maximum voluntary force; N, Newton; *M*_max,_ peak to peak amplitude of maximum compound action potential; EMG_max,_ maximum RMS EMG obtained during knee flexor maximum voluntary contraction.

Data are reported as mean ± SD (N = 12).

**Figure 2:**
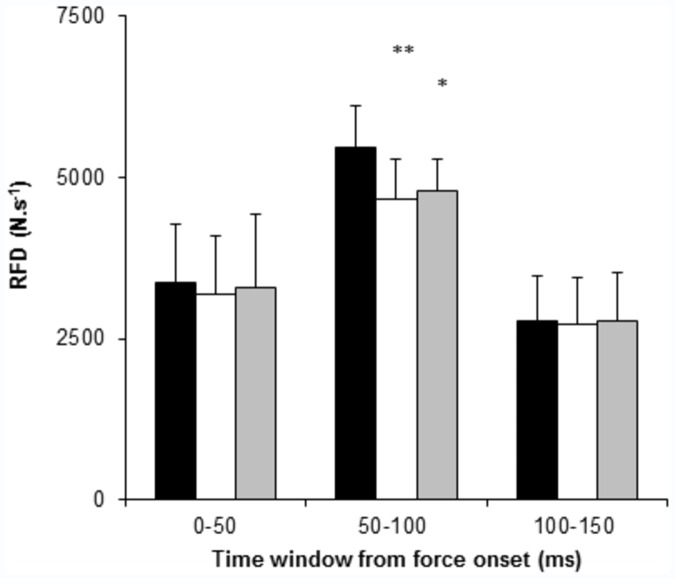
Rate of force development (RFD) during explosive unilateral (UL, black bars) and bilateral contractions (BL_BL_, white bars, averaged simultaneous performance of both limbs; BL_UL_, grey bars, single leg performance during BL contractions) explosive contractions of the knee extensors. Data are reported as mean (SD) (N = 12). A significant difference between conditions is denoted by *P<0.05 vs. UL, **P<0.01 vs. UL.

There was a significant difference between conditions for force (ANOVA, P = 0.022) and RFD (ANOVA, P = 0.022) during the explosive voluntary contractions. Pairwise comparisons revealed F_50_ was similar for all three conditions (P>0.90, Table 2). However, there was a BLD in F_100_ with BL_BL_ values 11.2% lower than UL (P = 0.007), and with a tendency for BL_UL_ to also be lower than UL (P = 0.067). There was a tendency for a BLD in F_150_ with BL_BL_ lower than UL (P = 0.059), but there was no difference in F_150_ between BL_UL_ and UL (P = 0.116, Figure 2). RFD_50­100_ was 14.9% lower for BL_BL_ (P = 0.004) and 12.5% lower for BL_UL_ (P = 0.022) compared to UL (Figure 2), with no differences in RFD_0­50_ or RFD_100­150_ between conditions (P>0.90). Additionally, there were no significant differences in RFD between BL_UL_ and BL_BL_ (All, P>0.90).

**Table 2:** Force during explosive voluntary contractions during unilateral (UL) and bilateral contractions (BL_BL_, averaged simultaneous performance of both limbs; BL_UL_, single leg performance during BL contractions).

Force (N)	UL	BL_BL_	BL_UL_
50 ms	168±45	159±46	165±57
100 ms	442±42	392±37**	404±56
150 ms	580±63	528±51	543±72

N, Newton; **denotes significant difference compared to UL (P<0.01).

Data are reported as mean ± SD (N = 12).

**Figure 3:**
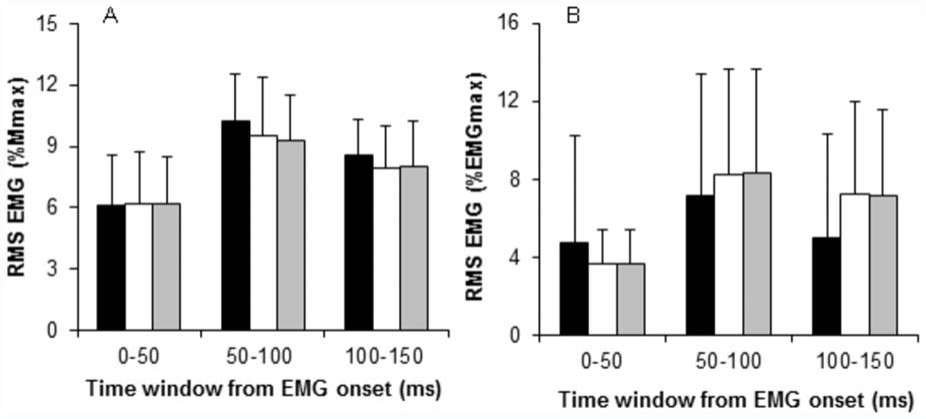
Agonist EMG normalised to *M*_max_ (A) and Antagonist EMG normalised to EMG_max_ during unilateral (UL, black bars) and bilateral contractions (BL_BL_, white bars, averaged simultaneous performance of both limbs; BL_UL_, grey bars, single leg performance during BL contractions) (B) explosive voluntary contractions. Data are reported as mean (SD) (N = 12).

There were no differences in agonist (two-way ANOVA, P = 0.233, Figure 3A) or antagonist (two-way ANOVA, P = 0.873, Figure 3B) EMG amplitude between the three measurement conditions during the explosive contractions. Additionally, neural efficacy, the percentage of evoked octet F_50_ achieved voluntarily was also similar for the three measurement conditions (UL, 55.5±17.3; BL_BL_, 58.4±18.7; BL_UL_, 61.3±20.6%, ANOVA, P = 0.212).

The time difference in force onset between the two limbs during the BL explosive contractions was 3.2±1.7 ms. There was no difference in EMD_max_ between UL and BL contractions (UL, 18.5±3.6 vs. BL_UL_, 18.4±4.1 ms, Paired t-test, P = 0.942). For BL_UL_, the best three contractions from each limb were taken for analysis irrespective of the performance of the other limb during that contraction. Of the 36 efforts taken forward for analysis in this condition (best three contractions, from each limb irrespective of the other limb for the 12 participants), 22 of them occurred within the same BL contraction.

### Electrically-evoked Contractions

Twitch F_50_ and PF were lower for both BL_UL_ and BL_BL_ compared to UL (7.8–9.1%, P≤0.002), with no difference for twitch pRFD between measurement conditions (Table 3). Additionally, there was no difference in *M*_max_ P-P between measurement conditions (UL, 3.0±1.1 vs. BL_BL_, 2.8±1.0 mV, Paired t-test, P = 0.138). Octet F_50_ was lower for both BL_UL_ (6.0%) and BL_BL_ (6.3%) than UL (Both, P<0.001, Table 3), but there were no differences for octet PF or pRFD (Table 3). There were also no differences between BL_UL_ and BL_BL_ for either twitch or octet measure (P≥0.187).

**Table 3:** Force parameters during evoked twitch and octet contractions during unilateral (UL) and contractions (BL_BL_, white bars, averaged simultaneous performance of both limbs; BL_UL_, grey bars, single leg performance during BL contractions).

	Condition:			
	UL	BL_BL_	BL_UL_	P-value
**Octet**				
F_50_ (N)	300±30	281±32	282±32	<0.001
PF (N)	480±52	477±55	480±54	0.585
pRFD (N.s^−1^)	13511±2785	13278±2433	14164±2892	0.243
**Twitch**				
F_50_ (N)	115±24	106±24	105±23	<0.001
PF (N)	134±27	122±26	123±24	<0.001
pRFD (N.s^−1^)	3920±1210	3709±1227	3754±1153	0.290

UL, unilateral; BL, bilateral; F, force; N, newton; PF, peak force; pRFD, peak rate of force development; F_50_, force at 50 ms after force onset. P-value, One-way analysis of variance significance value.

Data are reported as mean ± SD (Octet, N = 9; Twitch, N = 10).

## Discussion

This study investigated BLD in voluntary and electrically-evoked explosive contractions of the knee extensors and considered the contribution of agonist neuromuscular activation and measurement issues to any BLD. We observed a BLD in voluntary explosive force/RFD but not MVF. The BLD in explosive force occurred at 100 ms only and reflected a BLD specific to RFD_50­100_. BLD measurement issues made only a minor contribution to the observed BLD and thus these results support an underlying physiological mechanism explaining BLD. However, the fact that we observed a BLD in evoked force production and no change in EMG during explosive voluntary efforts suggests the BLD was not solely attributable to reduced agonist or antagonist neural drive.

The finding of no BLD in MVF is consistent with numerous reports (e.g. [10], [15], [24]), but in contrast to an equal number that have shown a BLD in knee extensor MVF (e.g. [2], [9], [25]). As there was no BLD in MVF, it is unsurprising that there was no difference in agonist or antagonist activation, evoked peak force measures with high force values, or influence of methodological factors. This is in accordance with previous findings of no BLD or mechanistic differences between BL and UL MVCs [1], [10].

Despite no BLD for MVF, we observed a BLD in explosive force of 11.2% during these single joint voluntary contractions. The BLD was specific to F_100_, but there was a tendency for a BLD in F_150_. Furthermore, there was a 14.9% BLD for RFD_50­100_, with no BLD for RFD_0­50_ or RFD_100­150_. This is the first study to investigate the possibility of a BLD in explosive strength by analysing force/RFD throughout the rising force-time curve. Previously, only pRFD had been assessed in this context, with BLD reported to range from 0–20% [3], [9], [10]. The mechanisms for the observed BLD in explosive force could have been due to measurement issues in the comparison of UL and BL performance, neuromuscular activation of agonist, antagonist muscles that were assessed in this study, or even activation of stabiliser muscles that we did not assess.

The assessment of single limb performance during BL contractions allowed for the delineation of measurement artefacts that may have contributed to any observed BLD. Although, the BL_UL_ measure reported only a tendency for a difference to UL for F_100_, there was a difference for RFD_50­100_, confirming a BLD due to a physiological effect exclusive of measurement issues. There were also no differences in explosive or maximal force/RFD between the two BL measures, indicating measurement artefacts played only a minor role in the observed BLD. Surprisingly, the onset of force discrepancy between the two limbs during BL contractions was relatively small (3.2 ms), which suggests that neuromuscular system is capable of near simultaneous activation of the knee extensor muscles of both legs during BL actions.

The current study found no differences in agonist EMG between UL and BL explosive contractions. This is despite the widely suggested mechanism for BLD being a reduction in neural drive to the agonist muscles. Our findings support previous research demonstrating a BLD in RFD in the absence of a change in agonist EMG [9]. It is important to note that the sensitivity of EMG for assessing BLD has been questioned [10]. However, in the present study we normalised the EMG amplitude to *M*_max_, which would be expected to increase the effect size and power of statistical comparisons between the conditions. Additionally, we averaged across three quadriceps muscles and across the best three contractions during the explosive efforts. These methods would be expected to improve the reliability and sensitivity of the EMG measurements. Furthermore, we also measured neural efficacy, which assesses agonist neuromuscular activation during the initial phase of the contractions (50 ms), and provided further evidence that agonist activation was not different during the early phase of UL and BL explosive contractions. These findings suggest that the observed BLD in RFD was not attributable to agonist activation, and indicates a role for an alternative mechanism.

Agonist and antagonist activation contribute simultaneously to net joint torque and thus the level of co-activation could account for any BLD. This is the first study to assess if antagonist activation influenced the BLD during explosive force production, and found that the observed BLD in RFD was not attributable to antagonist activation. A possible remaining explanation concerns stabiliser activation.

BL evoked contractions were utilised within the present study to help establish if the BLD was influenced by a physiological mechanism(s) exclusive of neural drive to the agonist muscles. Interestingly, there was a BLD in evoked force production, which occurred in both twitch and octet F_50_ (8.7 and 6.3%, respectively), and twitch PF (9.0%) and was of a similar magnitude to the observed declines in explosive voluntary force/RFD (8.6–14.9%). This is the first study to investigate a potential BLD in evoked force production and provides further support to the notion that the BLD in voluntary explosive force production was due to mechanisms other than agonist neural drive. A possible explanation for the BLD in both evoked and voluntary force is a difference in postural stability/stabiliser activation requirements during UL and BL actions. Stabiliser activation was not measured within the present study, but is thought to be important for optimal force expression [26]. For instance, Nozaki et al. [27] demonstrated that even during a relatively simple task such as an isometric knee extension used within the current study, that there was a large variation, both between and within-participants in the ability to stabilise the adjacent joint torque through effective inter-muscular coordination. The greater postural requirement for BL than UL strength tasks has been proposed as the mechanism accounting for the BLD in MVF [14]. In support of this suggestion, the BLD has been observed to be higher in an action requiring greater activation of postural stabilising muscles (leg press versus hand grip, [4]). In the current study insufficient stabilisation during BL explosive contractions may have afforded greater movement of adjacent joints, particularly the hips, increasing biological compliance and reducing explosive force production. Whilst the BLD in evoked explosive force we have observed might appear to contradict this possibility (as only the agonists are activated by the stimulation), there is undoubtedly stabiliser activation in anticipation of, and/or in response to, the stimulation, and this could be similarly less effective in the BL compared to UL situation. The similarity of MVF across BL and UL contractions might also argue against a role of stabiliser activation in the BLD we have observed, however, during these longer contractions force production is unlikely to be influenced by compliance and hence stabilisation. Future research should consider the role of stabiliser muscle activation in the BLD. The observed 15% deficit in RFD, despite no influence of BL actions on MVF has important implications for sport and exercise training science and suggests specific training to offset this deficit should be performed in order to maximise the performance of BL explosive sporting tasks. The observed deficit may have been explained by reduced inter-muscular coordination (lower stabiliser activation) during BL efforts and suggests that specific practice of coordinated explosive BL tasks and improved core/joint stability could be expected to improve the expression of BL explosive sporting tasks through reducing this explosive force/RFD BLD.

In summary, there was a BLD in explosive but not MVF of the knee extensors, which was specific to RFD_50­100_. Measurement artefacts not previously considered were shown to play only a minor role on the observed BLD confirming a BLD due to a physiological effect. The novel finding of a BLD in evoked force production and no change in agonist or antagonist EMG during explosive voluntary efforts suggest the BLD in voluntary explosive force may be attributable to changes in stabiliser activation.
